# A Brief Resilience-Enhancing Intervention and Loneliness in At-Risk Young Adults: A Secondary Analysis of a Randomized Clinical Trial

**DOI:** 10.1001/jamanetworkopen.2023.54728

**Published:** 2024-02-05

**Authors:** Nicole R. DeTore, Anne Burke, Maren Nyer, Daphne J. Holt

**Affiliations:** 1 Department of Psychiatry, Massachusetts General Hospital, Boston; 2 Department of Psychiatry, Harvard Medical School, Boston, Massachusetts

## Abstract

This secondary analysis of a randomized clinical trial assesses whether a behavioral intervention focused on resilience is associated with feelings of loneliness among young adults.

## Introduction

There has been a worldwide epidemic in loneliness for several decades,^1^ especially among adolescents and young adults. In one study, 40% of participants aged 16 to 24 years reported frequent feelings of loneliness.^2^ Addressing loneliness has become a public health priority as it has a well-established association with poor mental and physical health at all ages. In young people, loneliness has been associated with poor general health, physical inactivity, low sleep quality, increased substance use, and impaired psychosocial functioning.^3^ Loneliness is also a known risk factor for depression, anxiety, and suicidal ideation in young people. However, behavioral interventions focused on reducing loneliness have primarily targeted older adults and shown only moderate efficacy.^4^

As loneliness has been associated with negative self-evaluation and sensitivity to social rejection, we tested whether an intervention called Resilience Training (RT) focused on improving emotion regulation, self perception, and social interactions could lead to reductions in loneliness. The RT program is a 4-session, group-based behavioral intervention for young adults (aged 18-25 years), primarily college students, with mild risk factors for developing a psychiatric disorder (subclinical symptoms of psychopathology).^5^ It teaches evidence-based skills (mindfulness, self-compassion, mentalization) through didactic material, experiential exercises, and group discussions. In a previously reported randomized clinical trial,^5^ RT was found to significantly increase resilience-related capacities and reduce symptoms of psychopathology in at-risk college students. In this secondary ad hoc analysis of that trial, we tested whether RT was associated with reductions in loneliness.

## Methods

The procedures of the randomized clinical trial (NCT06038786) and this secondary analysis were approved by the Mass General Brigham institutional review board, and participants provided written informed consent for both the trial and this analysis. The study followed the CONSORT reporting guideline. The trial protocol is provided in Supplement 1.

Boston-area college students with mild depressive symptoms and/or subclinical psychotic symptoms (conferring an elevated risk for developing a psychiatric illness) were randomly assigned to receive RT (n = 54, 7-12 per group) or to a waitlist (n = 53) from July 2018 to February 2020. Participants completed the well-validated UCLA Loneliness Scale^6^ and other scales^5^ before and after the intervention period (Table). Self-reported race and ethnicity data were collected but not included as variables. A mixed-model analysis of variance was used to analyze the effect of RT on loneliness scores over time. This data analysis was performed in August 2023, using SPSS, version 29 (IBM) and a significance threshold of α = .05 (2-tailed), with an intent-to-treat design.

**Table.** Preintervention and Postintervention Changes in Loneliness and Resilience Measures by Intervention Group

Measure	Mean (SD) score					
	Resilience Training (n = 54)			Waitlist (n = 53)		
	Preintervention	Postintervention	Change	Preintervention	Postintervention	Change
Loneliness^a^	40.98 (12.11)	36.83 (11.69)	−4.27 (9.15)	39.37 (11.61)	40.62 (12.57)	1.46 (7.16)
Resilience^b^	64.94 (12.46)	72.70 (14.03)	2.45 (3.62)	55.89 (17.91)	60.48 (16.64)	0.44 (9.28)
Mindfulness^c^	124.44 (19.05)	132.38 (26.82)	6.73 (8.74)	110.53 (15.64)	115.53 (16.66)	1.33 (7.60)
Self-compassion^d^	2.91 (0.86)	3.45 (0.74)	0.32 (0.47)	2.65 (0.67)	2.82 (0.73)	0.03 (0.35)

^a^ Scores on the UCLA Loneliness Scale can range from 20 to 80, with higher scores indicating higher levels of loneliness.

^b^ Scores on the Connor-Davidson Resilience Scale can range from 0 to 100, with higher scores indicating greater emotional resilience.

^c^ Scores on the Five Facet Mindfulness Questionnaire can range from 0 to 195, with higher scores indicating a greater capacity for mindfulness.

^d^ Scores on the Self-Compassion Scale can range from 0 to 130, with higher scores indicating more self-compassion.

## Results

The study participants included 70 females (70%) and 30 males (30%) with a mean (SD) age of 18.8 (0.9) years, and 30 (30%) self-reported as Asian, 6 (6%) Black, 12 (12%) Latinx, 55 (55%) White, and 9 (9%) multiracial or other race and ethnicity. A significant group × time interaction (η^2^ = 0.11; *P* = .002) was found (Figure), indicating that RT participants experienced a greater decrease in loneliness than waitlist participants. Moreover, in the RT group, the preintervention to postintervention decrease in loneliness correlated significantly with increases in resilience (*r* = −0.42; *P* = .048), mindfulness (*r* = 0.06; *P* = .03), and self-compassion (*r* = −0.43; *P* = .051). No similar changes were found in the waitlist group. The group × time interaction for loneliness remained significant when controlling for changes in resilience (η^2^ = 0.19; *P* = .02) and mindfulness (η^2^ = 0.16; *P* = .04) but not for the increases in self-compassion (η^2^ = 0.10; *P* = .10), suggesting that the acquisition of this skill during RT contributed to the reduction in loneliness.

**Figure.**
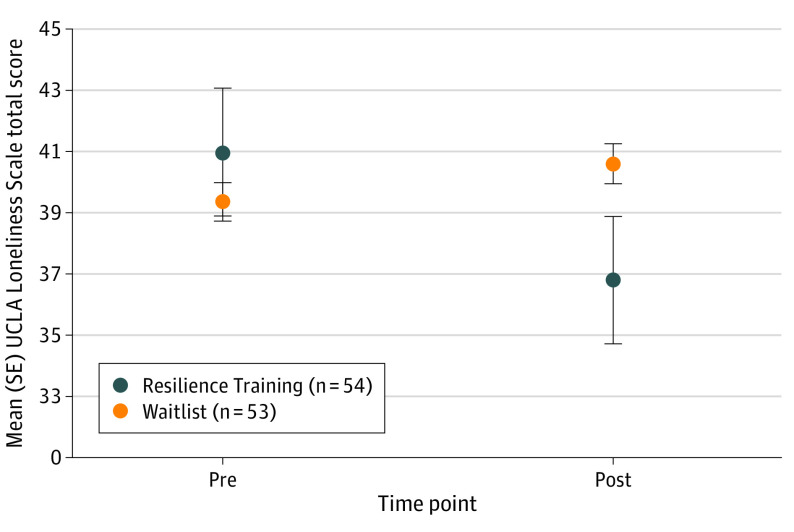
Loneliness in Each Group at the 2 Time Points A significant group × time interaction (η^2^ = 0.11; *P* = .002) was due to a greater decrease in loneliness in the group that received the 4-week Resilience Training intervention compared with the waitlist control group. SE indicates standard error.

## Discussion

These results suggest that RT may be an easily scalable tool for addressing and reducing the experience of loneliness among at-risk young adults. This study is limited by a lack of an active control group and follow-up assessments, which are needed to determine RT’s longer-term effects on loneliness, health, and well-being, and its key mechanisms, in college students and other populations.

## Notes

Supplement 1.
Trial Protocol

Supplement 2.
Data Sharing Statement
