# Supplementary material for: A Brief Resilience-Enhancing Intervention and Loneliness in At-Risk Young Adults: A Secondary Analysis of a Randomized Clinical Trial
Source: JAMA Netw Open. 2024 Feb 5;7(2):e2354728. doi: 10.1001/jamanetworkopen.2023.54728 (PMC10844993; doi:10.1001/jamanetworkopen.2023.54728)
Supplement: Supplement 1. — Trial Protocol [file jamanetwopen-e2354728-s001.pdf]

PROTOCOL SUMMARY

Title: Feasibility Testing and Waitlist-Controlled Randomized Controlled Trial of Resilience Training

Principal Investigator: Daphne Holt, MD, PhD

1) Background And Significance

Our group, the MGH Resilience and Prevention Program (RAPP) https://www.resilienceandprevention.com, has developed a brief behavioral intervention, called "Resilience Training" (RT), that is modeled on established, evidence-based treatments for treating psychiatric illnesses. The goal of RT is to help young people who may have one or more risk factors for psychiatric illnesses develop more effective coping skills for managing stress – particularly stress caused by challenging social interactions and negative emotions. RT is referred to as a "workshop" or "training", because it is focused on the *prevention* of psychiatric illness, rather than treatment of clinically established conditions. These trainings or workshops are administered by two clinicians (or a clinician and a clinician-in-training) to groups of 8-14 participants. In each session of RT, the leaders teach techniques that increase well-being and mindfulness and improve social functioning and emotion regulation capacity. The emphasis of the program is on enhancing existing strengths and coping skills (rather than on "fixing" deficits or "treating" symptoms), so that trainees are better able to weather the stresses and adversities that inevitably arise in people's lives.

Because youth with subthreshold symptoms of psychopathology do not typically seek treatment for their symptoms, it is necessary to develop new strategies for engaging this population in mental health-promoting behaviors. Interventions which are delivered in a convenient, non-burdensome manner, and focus on augmenting existing emotional strengths and inherent traits associated with emotional resilience (rather than focusing solely on reducing psychopathology) are greatly needed. Our team at RAPP has developed such interventions, which can be implemented in a variety of settings. The RAPP team has found that a training which focuses on social skills (i.e., capacities and behaviors, such as empathy, that are important for being effective in relationships and succeeding in one's school work and future career) and improving the ability to manage changes in emotional states in oneself and others, is well received by adolescents and young adults. The interventions are interesting and challenging and do not pathologize or stigmatize the participants. The overall importance (for all people) of learning and practicing resilience-enhancing skills for maintaining and improving emotional health, similar to the roles that exercise and healthy eating play in the maintenance of physical health, is emphasized. Consistent with this approach, in the college-based RT program, participants will be given the opportunity to provide the study team with feedback about the content of the workshop and suggestions for improving retention and participation. Each participant will be given a certificate at the completion of the program (which can be included on the participants' resumes) indicating that they participated in the program's design.

2) Specific Aims

The overall purpose of this program is to identify young people enrolled in college who endorse a range of severity of symptoms (mild-to-moderate) of subclinical psychosis and/or depression and provide them with a potentially preventive intervention: a 4-week resilience training (RT) workshop. Thus, with this program, our aims are the following:

> **Aim 1:** To measure the feasibility, sustainability and acceptability of an on-campus early detection and prevention program.

Aim 2: To measure the efficacy of the program in inducing short-term improvements in basic social processes (theory-of-mind capacity, personal space behaviors), social functioning (level of social activity, social satisfaction), resilience factors (ability to handle stress, self-efficacy) and symptom levels (attenuated psychotic symptoms, depression, and anxiety).

Aim 3: To measure the impact of the program on long-term outcomes (psychological, social, and school functioning).

Aim 4: To assess whether certain subject characteristics at baseline predict short-term and long-term outcomes.

The study includes 2 parts (each of which has a separate consent form):

Part A: A screening for eligible participants
Part B: The RT intervention

3) Subject Selection

Part A. Screening:
Inclusion criteria:
1. 18-25 years old.
2. Enrolled in undergraduate program at the college or university where the screening takes place.

Exclusion criteria:
1. Inability to provide informed consent.
2. Not proficient in English.

Part B. Intervention:
Inclusion criteria:
1. 18-25 years old.
2. Enrolled in undergraduate program at the college or university where the intervention takes place.
3. Students who endorse delusion-like experiences (DLEs): PDI score $\geq$ 4.
4. Students with mild to moderate depressive symptoms: BDI score $\geq$ 6.

Exclusion criteria:
1. Inability to provide informed consent.
2. Not proficient in English.
3. Current DSM-V diagnosis with active symptoms (such as active psychotic symptoms, current suicidality, serious active alcohol or substance use, marked deterioration in functioning over the prior month) determined by clinical interview with participant, or self-report of a psychiatric diagnosis that necessitates close monitoring or individual therapy and/or inpatient or partial hospitalization.
4. Current enrollment in psychological or behavioral health treatment.
5. Current use of psychotropic medications (other than stimulants) prescribed by a physician.

Source of subjects and recruitment methods
Subjects will be identified and referred to the study via: 1) bi-annual on-campus screenings (day-long events, or regular screenings of users of student health and other campus services) and 2) advertisements (self-referred).

The bi-annual screenings and the RT intervention will be conducted at the local university or college. Throughout the study, participants may be asked to complete questionnaires (before and after the RT intervention and during the longitudinal follow-up portion) through a RAPP-administered website.

4) SUBJECT ENROLLMENT

Enrollment in each part of the study (Part A and Part B) is implemented via two separate consent forms (Consent A and Consent B). The enrollment for Part A will take place at the on-campus screening, the enrollment for Part B will take place at the on-campus screening or before the first session of the RT intervention.

<u>Part A: On-campus screening:</u> A table or tent will be set up in a high traffic area of the school with signs posted nearby advertising for "free psychological," mental health," or "well-being" screening. The screening events will last approximately 8 hours per day for one or two days. Students who are interested will discuss the study with a member of the study team; if interested, the study team member will obtain consent.

<u>Part B: Intervention:</u> Students who complete Part A and are eligible for Part B may be informed they are eligible to participate in a workshop that focuses on honing leadership skills and developing new ways to cope with stress and challenges in interpersonal relationships.

In both consent forms, students will also be given the option to hear about additional studies being conducted at MGH. If interested in being contacted (by phone or email) to learn more information about these opportunities, the participant will opt in/out as noted in the consent form.

<u>Control Group:</u> After an initial piloting phase of the intervention, a waitlist control (WLC) group will be added to the study design. Half of the enrolled students (randomly selected during the pre-workshop/baseline assessment) will be selected for Group 1, which starts immediately following session B1, while the other half (Group 2) asked to wait for one round (approximately 4 weeks) before starting the RT intervention. The WLC students will complete the initial assessments at the same time as the students who will receive the RT intervention immediately and will also complete assessments before and after the 4-week intervention they complete. Thus, the WLC participants will undergo three assessments following the screening, rather than the two assessments (before and after the intervention) that the first group of students will complete. Although exclusionary criteria for Part B include current enrollment in psychological or behavioral health treatment, students in the WLC group will not be dissuaded from seeking treatment during the waitlist period, and eligibility for Part B will be reassessed prior to their enrollment in the intervention.

5) Study Procedures

<u>Part A: On-campus screening</u>

<u>Screening:</u>
Interested students will be given a consent form (**Consent form A**), discuss the consent form and receive answers to any questions with a member of the study team, and, if interested, that member of the study team will obtain informed consent. Consent form A also includes information about longitudinal follow-up questionnaires. These may be sent via an MGB secured link every six months. If the subject completes these questionnaires, they will receive additional compensation. They will also be able to grant permission for the study team to access and obtain information from their college records (including GPA, enrollment status, and use of behavioral health services). Academic information will be collected through the school longitudinally for students from Part A who grant permission in the consent form.

Also, some self-report academic information may be collected along with other questionnaires. Students will be made fully aware of these procedures during the consenting process. Students will also be asked to indicate and initial on the consent form if they are willing to be contacted at a later time regarding missing information that was not collected from the questionnaires administered at the screening. Students will be given a copy of Consent Form A to keep. After signing the consent form, the student may fill out the screening questionnaires and demographics form and later return them to the study team member for scoring.

Once informed consent has been obtained, the student will be asked to complete the set of questionnaires below. A member of the study team will score the BDI and PDI while the student is present, both to determine eligibility for the intervention (see Part B inclusion criteria above) and to determine whether an interview with an on-site member of the clinical study team (which may include licensed psychiatrists and clinical psychologists, psychiatry and clinical psychology fellows, and supervised psychology interns) is warranted. Psychology interns in our group receive weekly supervision from a staff psychologist; residents and fellows receive weekly supervision from a staff psychiatrist. Any trainee who becomes aware of a safety issue will contact his or her supervisor immediately.

If any student has concerning scores (scores that meet criteria for the interview), he or she will be asked to participate in the interview immediately.

An interview with a clinician will be conducted for enrolled students who have any of the following scores:

1. BDI total score ≥ 19 and a BDI #9 (the suicide item) score ≥ 1 or
2. BDI #9 score ≥ 2.

Prior research has shown that elevated symptoms of depression and/or endorsement of suicidality are associated with increased risk for suicide and other adverse events associated with acute psychiatric illness. An interview with a member of the clinical study team is thus indicated to allow the team to assess the risk level of the student. In accordance with clinical practice, if the member of the clinical study team determines there is clinically significant suicidality or other types of risk of harm to self or others on further evaluation, she or he will recommend appropriate steps, including contact with behavioral health services on the university campus. Participants will be considered to be at imminent risk of suicide if they have a plan to harm themselves in mind and current intent to execute a plan.

For individuals judged to be at elevated risk of suicide, we will ask the student to proceed to the nearest emergency room for a full evaluation. In the event they refuse care, and they are deemed by a member of the clinical study team to be at significant imminent risk for suicide, a psychiatrist or psychologist will fill out a request for involuntary hospitalization. Elevated risk for other types of self-harm (e.g., inability to care for self) or homicide will be treated in the same manner. If a student with concerning scores on the screening questionnaires declines the interview due to scheduling reasons (e.g., they need to get to class), we will encourage them to return for the interview portion later in the day. If a student declines the interview and is unwilling or unable to return at a later date, we will remind them that they can follow-up with any of the mental health resources we will provide all participants. If a student with particularly concerning scores (i.e., BDI #9 = 2 or 3) declines the interview, we will make three attempts to contact them in order to set up an in-person or phone interview. If we are unable to reach them or they decline the interview again, we will pass the student's information to our collaborating Universities, who will then attempt to establish contact with the student.

These risk management procedures follow successful risk management procedures used by other college-based studies conducted by MGH investigators.

Students may also be contacted if their screening questionnaires are missing important information, as long as they indicate that they are willing to be re-contacted in the consent form.

During the screening event, if eligible, students will be assessed for their interest in Part B (the RT intervention) portion of the study. Also, all students who participate in Part A will be offered a list of local mental health resources in the event that they wish to seek treatment outside of the study procedures. All students will be given a $20 gift card for their participation in the screening.

The following measures will be included in the assessment battery:

1. Demographic information
2. Family psychological health history measure
3. Self-report psychological health history measure
4. *Beck Depression Inventory (BDI)*, a 21-item questionnaire for the assessment of degree of depression*
5. *21-Item Peters et al. Delusions Inventory (PDI-short version)*, a 21-item questionnaire for the psychometric measurement of schizotypal traits and symptoms*
6. *State-Trait Anxiety Inventory (STAI)*, a 40-item questionnaire for the assessment of anxiety levels*
7. *Positive and Negative Affect Schedule* (*PANAS)*, a 20-item questionnaire for assessing positive and negative affect.
8. *Launay-Slade Hallucination Scale (LSHS)*, a 16-item questionnaire used to assess hallucinatory thoughts
9. *Childhood Trauma Questionnaire (CTQ-M*) a 47-item questionnaire for the assessment of childhood trauma
10. An adapted version of the "*Monitoring the Future" Substance Use Scale (MTF-2)* a survey asking detailed questions about subjects' use of 12 categories of substances.
11. *Perceived Stress Scale (PSS)*, a 10-item, widely-used psychological instrument for measuring the perception of stress.
12. *Interpersonal Reactivity Index (IRI)*, a 28-item questionnaire for assessing multiple dimensions of empathy.
13. *Social Network Index Adapted for College Students (SNI-adapted)*, a 12-item questionnaire used to assess the size and diversity of an individual's social network.
14. *Chapman Social Anhedonia Scale revised (SAS)*, a set of 40 true-false questions used to assess the level of pleasure experienced from and importance assigned to social interaction.
15. *Time Alone Questionnaire (TAQ)*, a 6-item questionnaire used to assess the portion of waking hours spent with others, compared to desired amount of time spent with others.
16. *Psychological Well-Being Midus-1 Version (PWB)*, an 18-item questionnaire for assessing several qualities of overall well-being.
17. *UCLA Loneliness Scale, a* 20-item scale to measure one's subjective feelings of loneliness.
18. *Self-Compassion Scale (SCS)*, a 26-item scale for the assessment of self-compassion.
19. *General Self-Efficacy Scale (GSE)*, a 10-item questionnaire for the assessment of self-efficacy, the ability to cope with stressors.
20. *Five Facet Mindfulness Questionnaire (FFMQ)*, a 39-item questionnaire that assesses the 5 domains of mindfulness.
21. *Connor-Davidson Resilience Scale (CD-RISC)*, a 25-item questionnaire for assessing resilience.
22. *Aberrant Salience Test* (AbSal): a list of 108 words to be rated as neutral or negative, designed to measure negative attribution bias.
23. *Paranoia Checklist – 5-item* (PC-5): a 5-item measure of symptoms of paranoia.
24. *Mentalization Scale (MentS)*, a 28-item questionnaire to assess mentalization.

**primary outcomes, predicted to decrease in magnitude following RT but not following the WLC condition.*

Part B: Intervention
This portion of the study contains 6 visits: B1 (the pre-enrollment session), visits B2-B5 (the RT intervention), and visit B6 (post-intervention session). Students will be compensated $20 via check for each visit during Part B, up to $120 total or $140 if selected for the waitlist control group, for which students earn an extra $20 for the extra session of assessments.

Pre-Workshop/Baseline Session:
At this session, if not already done so in Part A, the students will receive Consent form B and discuss the consent form and answer any questions posed by the participants and provide more information concerning the intervention, and, if interested, that member of the study team will obtain informed consent. The subject will then receive a copy of the consent form.

The previous assessments will be administered again during visit B1 with the addition of the *Knowledge and Feasibility Scale of RT intervention.*

If there is less than one month in between the screening event and visit B1, the study team will use the questionnaires answered during the screening event and only administer scales that were not completed at the screening event or are only valid for periods of time shorter than one month, i.e., the BDI, STAI – state, and PANAS.

The waitlist control group will complete this round of assessments with the group held immediately after the screening, then they will repeat it once the waitlist period is over.

RT Intervention:
This intervention is comprised of techniques derived from established treatments of psychiatric illnesses including: mindfulness, Cognitive Behavioral Therapy (CBT), Mindful Self-Compassion (MSC) therapy and Mentalization Based Treatment (MBT). The RT intervention begins with an introduction to the concepts of resilience and mindfulness; mindfulness techniques are taught and emphasized throughout the program. The RT intervention consists of four (4) weekly sessions. Each intervention group will have approximately 8-14 participants.

The intervention combines these modalities during the course of the four sessions, in the following manner: The first session will introduce the concept of resilience and mindfulness and the practice of mindfulness; the second session will then introduce MSC concepts and skills; the third and fourth sessions will introduce mentalization skills and integrate all of the modalities, using simulated (film clips) and real life examples provided by participants, to facilitate the implementation of the concepts and skills in everyday life. Participants will also be given short homework assignments after each session to help them use the skills and understand their benefits.

Intervention materials may be provided to the participants during the workshops to enhance their learning, however, no additional assessments will be administered during the four sessions of the RT intervention.

In order to assess and maintain fidelity to the intervention manual across iterations of the intervention, the leaders of the group may audio record each intervention session. Members of the clinical study team who have not led the intervention will then listen to a portion of these recordings and rate the sessions based on predetermined adherence criteria. Subjects will be informed of the potential for audio recordings of sessions for only this purpose prior to consenting to Part B. Recordings and recording devices will be stored behind locks and/or encryption in order to ensure subject privacy. Fidelity ratings will be made according to the RT Adherence Measure.

PROTOCOL SUMMARY

Post-Intervention Session:
Following the conclusion of the 4 weekly workshop sessions, the members of the group will be scheduled for an additional individual meeting, during which each student will meet with members of the study team. They will discuss ways in which the intervention succeeded and ways that it could be improved. The same assessment battery will be used in Visit B6.

Longitudinal Follow-Up:
Participants who completed Part A of the study will be asked to complete online questionnaires every six months for up to two years following the intervention. If they agreed to the collection of academic information in Consent A, a member of the study team will work with the local college/university to obtain this information. Participants will be mailed $10 in the form of a check for each online follow-up assessment they complete. The assessment will be the same as the initial survey battery. A member of the study team will review the participants' scores for potential safety concerns within 24 hours following a survey submission.

If a participant indicates mild suicidal ideation (BDI #9 Suicide Item = 1, which is endorsed by 10-15% of the college student population nationwide) and moderate depressive symptoms (BDI total score = 19-29), Dr. Holt, the PI of the study, will be notified, and a member of the study team will reach out to that participant via phone and/or email in order to provide an assessment via phone or in person. See below * for potential outcomes and actions that may follow this assessment. At minimum, the study team member will provide the student with a list of mental health resources and discuss with the participant a plan in case their symptoms worsen.

In the event the student endorses 2 or higher on the BDI #9 Suicide Item (indicating suicidal ideation with intent) or a 1 on the BDI #9 item and severe depressive symptoms (BDI total score > 29), Dr. Holt, the PI of the study, will be notified, and a member of the clinical study team will contact the student via phone and/or email in order to provide an assessment via phone or in person. In the event that the student does not respond, the University will be informed about the scores of the student and may contact them.

* In accordance with clinical practice, if the study clinician determines there is clinically significant suicidality on further evaluation, they will recommend appropriate steps. This may range from contacting other treaters, if any; arranging for an urgent appointment at the University or College counseling program; or referring the student to a local emergency service such as the Acute Psychiatry Service at MGH. Depending on the nature/level of the risk and the student's ability to comply with recommendations, the study clinician may also need to issue a Section 12 that authorizes emergency transport to and evaluation in an appropriate facility. Study participants will be informed of the risks associated with study participation including the possibility that in the event that there is concern about a serious risk of suicidality (or harm to others), participants' private information may need to be shared in order to ensure his or her safety. These procedures are clearly described in the consent forms.

For academic information, participants must sign a separate section of Consent form B. This is asking for permission to obtain, in collaboration with each university, academic information for the participants. All information obtained through the school registrar and counseling center. The following measures will be obtained via the college of enrollment every 12 months for those who have given written permission.

1. GPA
2. Use of mental/behavioral health services
3. Enrollment status, including leaves of absences and withdrawals from school

Research Electronic Data Capture (REDCap):
For the online follow-up assessments, students will receive an individual secure link (with a unique username to enter their data) to the questionnaires on REDCap (Research Electronic Data Capture) every

six months for two years following the screening. The online follow-up assessments will begin within two months after the completion of the RT intervention. These self-report measures will be collected using REDCap (Research Electronic Data Capture), a platform for electronic data capture that streamlines data collection and management, and ensures data integrity, resulting in improved data quality. The REDCap software allows researchers to design and implement study surveys for collecting, storing, retrieving, and manipulating data electronically. Participants will enter survey responses into electronic assessment forms, and the responses are then transmitted securely via encrypted connection and stored in a secured database. This electronic data capture eliminates the need for subsequent data entry by staff, thus minimizing human error. For this study, participants will complete the electronic assessments on their own computer terminals, under specific usernames sent by the Research Assistant in individually addressed e-mails. Using 128 bit RSA Secure Socket Layer (SSL) encryption, pre-determined users will log in to the questionnaires using secured browsers behind the MGB Healthcare Systems IS corporate firewall. Participants will be e-mailed a unique identifying key that will link them to various electronic forms. Therefore, each student/subject will only have access to his or her own survey data.

Data will be stored automatically and securely on an MS SQL Server, accessed over industry standard SSL 128 bit RSA encryption during data transfers. Data is routinely backed up locally onto a redundancy server and stored in a separate database. Long term storage on MGB servers occurs nightly and allows for incremental backup over multiple systems. Therefore, should one drive be physically damaged, there will be multiples within the chain to replace it. Both data servers are stored within MGB IS corporate firewall, in a secure, key access facility with password-protected computers. Only vetted MGB IS security officials will have access to physical machines storing study data. Since data are stored on a protected server, a compromise of any individual computer at a research facility will not lead to a breach of the secure database. Individual computers designated for data capture do not store participants' identifying information or study data.

6) Biostatistical Analysis

To measure the feasibility and acceptability of the prevention program (Aim 1), we will conduct qualitative and quantitative analyses of the recruitment and retention of participants to the RT Intervention. We will report descriptive statistics of those who participated in the RT group and compare them to those who were offered the group and declined. In addition, we will conduct analyses of the knowledge, feasibility, and acceptability measures to determine whether the RT Intervention increased participant's knowledge base of resilience-promoting tools and behaviors. To measure the efficacy of the program (Aim 2), we will conduct repeated measures analysis of variance (ANOVAs) to determine whether the RT Intervention leads to improvement of symptoms (i.e., psychotic experiences, depression and anxiety, the primary outcomes), social functioning/processing, and resilience, compared to the WLC condition. In addition, we will utilize linear and logistic regression to examine potential moderators to treatment response. To measure the impact of the program on long-term outcomes (Aim 3) we will conduct linear regressions using a dichotomous responder variable to regress on the psychological, social, and school functioning variables over time. Further exploratory longitudinal analysis will be conducted with hierarchical linear regression (HLM) to examine person-level variables that may impact long-term outcomes. Finally, multiple regression analyses will be conducted to identify predictors of symptom change and long-term outcomes (Aim 4).

7) Risks And Discomforts

Participation in the screening, intervention and follow-up may produce psychological discomfort. Some participants may find completing questionnaires to be burdensome and/or emotionally painful. The RT intervention could be considered time consuming. It also may be difficult for some students to talk about

themselves in a group-based setting. Participants receiving the intervention may not experience improvement in mental health. During the follow-up phase, filling out follow up questionnaires online may also be considered time consuming. No other risks or discomforts are expected. Although confidentiality will be strictly maintained, participants will be informed that the investigators have an obligation to take all necessary and clinically appropriate steps to ensure safety, in the rare event that safety concerns arise.

8) Potential Benefits

There may be no direct benefit from study participation. However, there are possible benefits for both study participants and society from this research. *For screening participants*, they may learn more about their mental health. *For those who participate in the RT intervention*, they may experience an improvement in their coping mechanisms and overall resilience due to the intervention. *For those who participate in the follow-up component of the study*, they may benefit from the ongoing self-monitoring of their symptoms and functioning. *For society*, such benefits may include increased knowledge about early detection of mental health problems, and the prevalence of subthreshold and clinical psychosis, depression and anxiety in the undergraduate student population.

9) Monitoring And Quality Assurance

Subjects will be monitored closely throughout the course of their study participation. If the PI or study clinician determines that a subject's continued participation is unsafe or not in their best interest, they may be terminated from the study. Confidentiality will be maintained by assigning a unique study ID for each participant. All data will be stored in a locked filing cabinet and/or password protected computer. Only IRB approved study team members will have access to the data. Any data containing protected health information (PHI) will be stored separate from de-identified data.

Quality assurance procedures will include training of all study personnel (including psychology/psychiatry trainees) prior to initiation of the study and monitoring of subject enrollment and ensuring quality of data (source documentation and online in RedCap). Internal audits will be conducted monthly by the Sr. Program & Research Manager, under direct supervision of the PI, to assure that study data in RedCap are accurate and in agreement with source documentation, verify that consent for the study has been properly obtained and documented, and confirm that participants enrolled in the study meet inclusion and exclusion criteria. Any significant deviations from the protocol, including non-adherence to inclusion/exclusion criteria, errors in study procedures, or failure to complete assessments, will be reported to the MGB HRC as a protocol violation.

The PI will be responsible for ensuring timely reporting of all unanticipated problems and adverse events experienced by the participant in accordance with the MGB HRC guidelines. The HRC guidelines in the following *unanticipated problems* and *adverse events* include incidents that occur: 1) during the conduct of the study, 2) after study completion, or 3) after subject withdrawal or completion. Reports are to be submitted within 5 working days or 7 calendar days of the date the investigator first becomes aware of the problem.

Reports of *unanticipated problems involving risks to subjects or others* are to be submitted through Insight/eIRB within 5 working days or 7 calendar days of the date the investigator first becomes aware of the problem.

Reporting Unanticipated Problems that are Adverse Events

Any unanticipated, untoward, or unfavorable medical occurrence that indicates that the research places subjects at increased risk of physical or psychological harm than previously known or recognized are to be submitted through Insight/eIRB as an Other Event, Adverse Event.

Reporting Unanticipated Problems that are not Adverse Events

All other unanticipated incidents, experiences, information, outcomes, or other problems that indicate that the research places subjects at an increased risk of physical, psychological, economic, legal, or social harm than was previously known or recognized are to be submitted through Insight/eIRB as an Other Event.
