# Supplementary material for: A Brief Resilience-Enhancing Intervention and Loneliness in At-Risk Young Adults: A Secondary Analysis of a Randomized Clinical Trial
Source: JAMA Netw Open. 2024 Feb 5;7(2):e2354728. doi: 10.1001/jamanetworkopen.2023.54728 (PMC10844993; doi:10.1001/jamanetworkopen.2023.54728)
Supplement: Supplement 2. — Data Sharing Statement [file jamanetwopen-e2354728-s002.pdf]

Data Sharing Statement

DeTore. Effect of a Brief Resilience-Enhancing Intervention on Loneliness in At-Risk Young Adults. *JAMA Netw Open*. Published February 05, 2024.
doi:10.1001/jamanetworkopen.2023.54728

Data
Data available: Yes
Data types: Deidentified participant data
How to access data: https://data.mendeley.com
When available: With publication

Supporting Documents
Document types: None

Additional Information
Who can access the data: It will be open to anyone requesting the data
Types of analyses: For any purpose
Mechanisms of data availability: Without investigator support
